# Service Quality Assessment of Digital Health Solutions in Outpatient Care: Qualitative Item Repository Development Study

**DOI:** 10.2196/68276

**Published:** 2025-07-24

**Authors:** Dominik Rigo, Leonard Fehring, Achim Mortsiefer, Sven Meister

**Affiliations:** 1 Health Care Informatics, Faculty of Health School of Medicine Witten/Herdecke University Witten Germany; 2 Department of Gastroenterology Helios University Hospital Wuppertal Wuppertal Germany; 3 General Practice II and Patient-Centredness in Primary Care, Institute of General Practice and Primary Care, Faculty of Health School of Medicine Witten/Herdecke University Witten Germany; 4 Department Healthcare Fraunhofer Institute for Software and Systems Engineering ISST Dortmund Germany

**Keywords:** digital health solutions, health care service quality, patient satisfaction, health service outcomes, implementation outcomes, health technology assessment

## Abstract

**Background:**

The integration of digital health solutions (DHSs) into health care systems has the potential to significantly enhance service delivery and health outcomes. Despite their benefits, the adoption remains slow, especially in outpatient care, and is hindered by various barriers, such as unclear effectiveness and high costs.

**Objective:**

This study aimed to address the uncertainties regarding the cost-benefit ratio of DHSs by developing a comprehensive instrument to evaluate their impact on health care service quality across diverse settings (eg, across different diseases or types of DHSs).

**Methods:**

We conducted a multistaged rapid review and semistructured, qualitative interviews to identify and adapt existing instruments evaluating the effects of DHSs. The first rapid review screened 4957 records and included 40 relevant papers to identify instruments currently used for DHS assessment after their deployment, yielding a total of 126 reported outcomes. Subsequently, we conducted interviews with 19 health care practitioners across 4 countries to validate and refine the 7 health care service quality dimensions derived from merging the Outpatient Experience Questionnaire (OPEQ), selected after the first rapid review, and Health Care Service Quality (HEALTHQUAL), an established instrument for measuring health care service quality derived from gray literature. On the basis of the results of the interviews, a second rapid review with 35 papers out of 493 screened records was conducted to identify instruments used to measure patient satisfaction, yielding a total of 29 patient satisfaction instruments.

**Results:**

From the first rapid review, OPEQ was selected out of 18 relevant instruments identified among the 126 reported outcomes and combined with HEALTHQUAL. The interviews with health care professionals confirmed the relevance of all 7 health care service quality dimensions derived from OPEQ and HEALTHQUAL. In addition, 4 interviewees mentioned patient satisfaction as a further dimension missing in the framework presented during the interviews. From the subsequent rapid review, the Patient Satisfaction Questionnaire-Short Form was selected out of 6 relevant instruments identified among the 29 identified patient satisfaction instruments. By combining HEALTHQUAL, OPEQ, and Patient Satisfaction Questionnaire-Short Form, we derived the Digital Healthcare Service Quality (DigiHEALTHQUAL) questionnaire, which consists of 51 items across 8 dimensions, including accessibility, efficiency, empathy, general satisfaction, degree of improvements of care services, information, safety, and tangibles.

**Conclusions:**

The DigiHEALTHQUAL questionnaire aims to provide a standardized approach for assessing the impact of DHSs on health care service quality across various use cases, therapeutic areas, and perspectives, facilitating comparison between DHSs and supporting decision makers in resource allocation and implementation decisions. Future research will focus on validating the DigiHEALTHQUAL in real-life settings and further refining it to comprehensively encompass both patient and health care practitioner perspectives.

## Introduction

### Theoretical Background

The integration of digital health solutions (DHSs) into health care has become an area of extensive research [1,2]. For our study, we adopted the European Commission’s definition of DHSs, which describes them as “tools and services that use information and communication technologies to improve prevention, diagnosis, treatment, monitoring and management of health-related issues and to monitor and manage lifestyle-habits that impact health” [3]. The many ways in which DHSs can transform health care have been shown in various studies, such as achieving cost and time savings [4], improving medical safety and treatment adherence [5], enhancing service quality [6], and ameliorating health outcomes [7].

Despite these benefits, the adoption of DHSs in health care is slow [8]. This is even more pronounced in outpatient settings compared to inpatient settings [9,10]. The key obstacles include required workflow adjustments, high costs for installation or maintenance, and unclear or limited effectiveness [11,12].

These challenges, especially the considerations between effectiveness and costs, underscore the importance of reliably measuring the impact of DHSs. Reliable measurement can support the further adoption of DHSs, that is, by helping decision makers compare the effects of different DHSs, select the most suitable DHS for their organization, and monitor the progress of deployments [12-14].

Moreover, as the landscape of DHSs grows increasingly complex with more and more digital use cases, tools, and providers [15], it is becoming more difficult for decision makers to navigate this landscape. Therefore, robust evaluation frameworks become more important for their support [16]. Recent literature reveals that the instruments currently used to assess the implementation of DHSs often lack robust psychometric properties, leading to a lack of consensus on preferred assessment methodologies [17-19]. Existing validated instruments tend to focus on specific use cases or diseases, which limits their utility for comparison across multiple DHSs and health care settings [20-22]. This variation in quality and scope of reported effects complicates the task for decision makers attempting to compare, select, and implement DHSs.

### Objectives

To address the limitations of currently applied instruments, it is essential to develop and establish comprehensive instruments. Existing studies have used a range of broadly applicable instruments, for example, measuring costs, resource use, patient satisfaction, and quality of life [20]. For example, the Mobile Healthcare App Database uses the Mobile Application Rating Scale to increase transparency regarding the quality of content and data security of >1300 DHSs [23,24].

Although service quality is a well-established and extensively studied outcome in various sectors, including health care—with numerous validated instruments available [25]—to our knowledge, established instruments for health care service quality (HCSQ) evaluation have not yet been used to assess the effects of DHSs. Recognizing the necessity for a versatile outcome measure, we considered whether HCSQ might be an effective metric to assess the effects of DHSs in a variety of health care settings. Therefore, this study aimed to address the lack of broadly applicable and reliable instruments by identifying, adapting, or developing a questionnaire that can measure the service quality of DHSs across different settings leveraging existing instruments. The broad adoption of such a tool would be particularly valuable for decision makers when considering the selection and implementation of DHSs.

## Methods

### Overview

**Figure 1:**
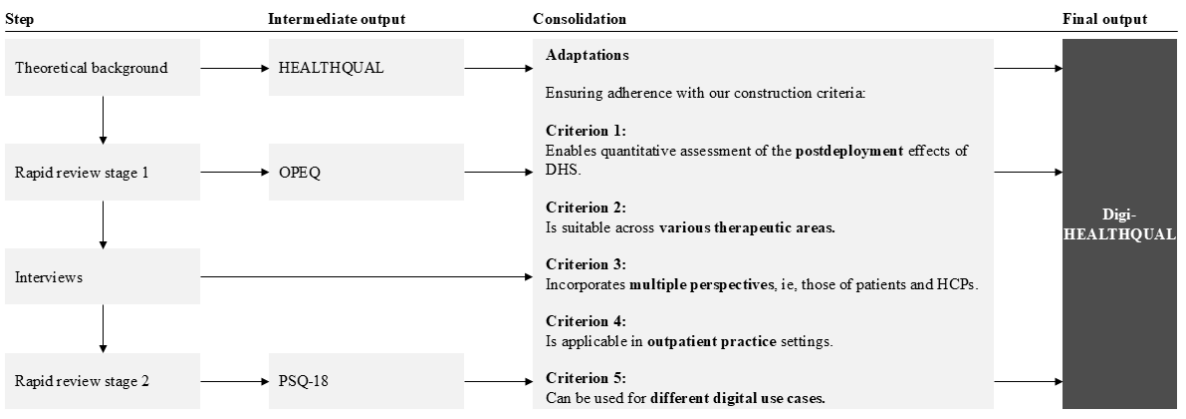
Instrument consolidation and adaptation strategy for the development of Digital Healthcare Service Quality (DigiHEALTHQUAL). The stepwise approach included the development of the theoretical background and identification of Health Care Service Quality (HEALTHQUAL), a first rapid review identifying Outpatient Experience Questionnaire (OPEQ), interviews with health care practitioners (HCPs), and a second rapid review identifying Patient Satisfaction Questionnaire-Short Form (PSQ-18). Consolidation steps ensured alignment with 5 construction criteria: postdeployment applicability, cross-therapeutic relevance, multistakeholder perspectives, outpatient care focus, and applicability across digital use cases. DHS: digital health solution.

This study used a sequential approach (Figure 1) following predefined construction criteria to create a comprehensive questionnaire that can measure HCSQ in settings with different DHSs, across therapeutic areas, and from both patient and health care practitioner (HCP) perspectives for the assessment of the effects of DHS deployment on HCSQ.

We selected Health Care Service Quality (HEALTHQUAL) [26]—a validated, health care–specific service quality instrument based on the well-established service quality model Service Quality (SERVQUAL) [27]—for further adaptation to the context of this study. As HEALTHQUAL has not previously been used in digital health settings, we conducted a rapid review adhering to the PRISMA (Preferred Reporting Items for Systematic Reviews and Meta-Analyses) guidelines [28], as well as recommendations from Tricco et al [29] and King et al [30]. This review helped identify existing instruments used to assess the postdeployment effects of DHSs. The Outpatient Experience Questionnaire (OPEQ) was selected for the subsequent steps [31,32].

Neither the original HEALTHQUAL nor OPEQ have been validated for use with HCPs. To address this limitation, we conducted semistructured qualitative interviews with 19 HCPs, following the COREQ (Consolidated Criteria for Reporting Qualitative Research) checklist [33]. This process validated the relevance of the included dimensions for HCPs and led to the addition of a dimension for patient satisfaction, which was derived from the Patient Satisfaction Questionnaire-Short Form (PSQ-18) after an additional literature review on patient satisfaction measurement instruments [34].

Consequently, we derived our suggested set of dimensions and items, hereafter referred to as Digital Healthcare Service Quality (DigiHEALTHQUAL).

### Construction Criteria for the Questionnaire

To identify, adapt, or develop a questionnaire, we defined the following five criteria (Figure 1):

1. Enables quantitative assessment of the postdeployment effects of DHSs following the definition of Proctor et al [35] for implementation outcomes
2. Is broadly applicable and suitable across therapeutic areas
3. Incorporates multiple perspectives, that is, those of patients and HCPs, including health professionals, health associate professionals, and health management and support personnel, as defined by the World Health Organization (WHO) [36]
4. Is applicable in outpatient practice settings, following the definition provided by the National Cancer Institute [37]
5. Can be used for different digital use cases, that is, those already prioritized for the next research stage following this work (online appointment booking platforms, digital anamnesis tools, video consultations, and electronic patient records)

### Selection of Research Framework

One well-established tool in the area of health service is SERVQUAL [27], which is widely used to measure service quality across different sectors [38]. SERVQUAL has often been adapted to measure HCSQ in various health care settings [25,39]. This led to the development of several health care–specific variations of SERVQUAL. The 3 frequently used variations are called HEALTHQUAL and were developed by Miranda et al [40], Lee [26], and Mosadeghrad and Sokhanvar [41]. Despite evidence that DHSs impact HCSQ [1,6], none of these HEALTHQUAL instruments have been used to assess these effects. Therefore, this study aimed to fill this gap by adapting HEALTHQUAL by Lee [26] to evaluate the impact of DHSs on HCSQ.

For this study, we used HEALTHQUAL by Lee [26] because of its comprehensive design approach, which incorporated input from experts and patients as well as international health care service accreditation systems. HEALTHQUAL by Lee [26] closely aligns with the original SERVQUAL framework and adds a dimension for health outcomes, making it a robust tool for assessing HCSQ [26]. In addition, HEALTHQUAL by Lee [26] has been widely applied internationally in both inpatient and outpatient settings (eg, South Korea, India, Syria, and Ghana) [42-47]. The instrument consists of 5 dimensions with a total of 32 items. These dimensions are empathy, tangibles, safety, efficiency, and the degree of improvements of care services.

### Multistaged Rapid Review

#### Overview

We conducted 2 rapid literature reviews following the PRISMA-RR (Preferred Reporting Items for Systematic Reviews and Meta-Analyses Extension for Rapid Reviews) guidelines and recommendations for rapid reviews outlined by Tricco et al [29] and King et al [30]. As PRISMA-RR is still under development, we adapted PRISMA (Preferred Reporting Items for Systematic Reviews and Meta-Analyses) guidelines (especially PRISMA-ScR [Preferred Reporting Items for Systematic Reviews and Meta-Analyses Extension for Scoping Reviews]) to align with the scope of this project. The search strategy and selection criteria are detailed below. More details can be found in Multimedia Appendices 1 and 2. Owing to the larger number of abstracts retrieved after title screening in the rapid review stage 1, we used ASReview LAB (Zenodo), an artificial intelligence–assisted screening tool, to expedite the abstract screening process [48]. This step was terminated upon meeting predefined stopping criteria in line with recent literature [49,50], specifically after screening at least 50% (422/843) of the abstracts and encountering at least 50 consecutive irrelevant abstracts. Data from the included studies were synthesized by extracting details from the reported findings and the instruments applied. This information was used to select suitable instruments for the following instrument development.

#### Rapid Review Stage 1

##### Overview

We conducted our search across 3 databases: PubMed, Scopus, and APA PsycINFO. Our search used a structured string comprising 4 conceptual blocks: *digital health, implementation, effects,* and *assessment*. The search terms were adapted from recent reviews within this field [1,7,17,19,51-60]. Articles were selected according to the criteria described in the following sections.

##### Inclusion and Exclusion Criterion 1: Type of Article

The articles were included if they reported the outcomes of a comprehensive data collection and analysis process. The articles were excluded if they (1) focused on describing the study design or methodology, (2) were statements, (3) reported single case studies, or (4) were conference papers.

##### Inclusion and Exclusion Criterion 2: Reported Outcomes

The articles were included if they (1) reported quantitative measures to assess postdeployment effects of DHSs and (2) included outcomes beyond health-related outcomes. The articles were excluded if they (1) focused on qualitative methods, (2) focused on intervention development and implementation, (3) focused on health-related outcomes, or (4) focused on describing the current status and trends of the health care sector in this area.

##### Inclusion and Exclusion Criterion 3: Type of Use Cases

Articles were included if they described the postdeployment outcome of a DHS that (1) belonged to 1 of the 4 selected digital use cases, (2) was implemented in an outpatient setting, and (3) did not require specific hardware. Articles were excluded if (1) the articles did not describe a DHS within the defined scope, (2) the DHS was deployed in an inpatient setting, or (3) the DHS required specific hardware.

#### Rapid Review Stage 2

##### Overview

We limited our search to the PubMed database after observing in rapid review stage 1 that it yielded the most relevant results for our research. Our search used a structured string comprising 3 conceptual blocks: *patient satisfaction, assessment,* and *primary health care*. The articles were selected according to the criteria described in the following sections.

##### Inclusion and Exclusion Criterion 1: Type of Article

The same criteria applied in rapid review stage 1 were used here to determine eligible article types.

##### Inclusion and Exclusion Criterion 2: Reported Outcomes

The articles were included if they contained patient satisfaction measures as at least 1 of the reported findings. The articles were excluded if they did not include a patient satisfaction measure.

##### Inclusion and Exclusion Criterion 3: Reported Health Care Setting

The articles were included if the study was conducted in a primary health care setting. The articles were excluded if the study was conducted in a different setting.

##### Inclusion and Exclusion Criterion 4: Reported Instruments

The articles were included if patient satisfaction was assessed using a validated instrument or if they referenced another publication as the source for the instrument. The articles were excluded if the applied instrument was (1) self-constructed, (2) not validated, or (3) did not reference previous scientific work.

### HCP Interviews

We conducted qualitative, semistructured interviews with 19 HCPs. These interviews followed the COREQ checklist [33]. More details can be found in Multimedia Appendix 3.

We designed an interview guide to validate findings from rapid review stage 1 and to explore additional HCSQ dimensions relevant to HCPs. More details are provided in Multimedia Appendix 4. The interviews focused on two domains: (1) implementation and current experience with DHSs and (2) evaluation and further exploration of HCSQ dimensions.

Interviewees were recruited through targeted outreach to both contacts within the researchers’ network as well as to HCPs with relevant profiles and publicly available contact information. The following profiles were included: (1) working as an HCP [36], (2) working in outpatient care [37], and (3) considering the implementation or already using at least 1 of the selected DHS use cases. We ensured a diverse sample across specialties, roles, demographics, and countries.

The interviews were conducted with each interviewee individually over the phone. Each session was audio recorded and transcribed verbatim. Recruitment concluded after 19 interviews, upon reaching data saturation. The average duration of each interview was 29 (SD 8) minutes.

We performed coding and qualitative analysis using MAXQDA software (Verbi software) [61]. Our content analysis approach combined deductive and inductive methods [62]:

- Deductive coding established codes based on our rapid review stage 1 results to facilitate comparison.
- Inductive theme derivation derived additional themes inductively from the interview content if mentioned by >1 interviewee.

On the basis of these analyses, we quantitatively scored the relevance of each literature-derived dimension.

### Ethical Considerations

The study protocol was reviewed and approved by the Ethics Committee of Witten/Herdecke University (S-113/2023). All interviewees received written information outlining the study’s objectives, procedures, and data handling practices and provided written informed consent before participation. Participation was entirely voluntary, and no incentives were offered. Audio recordings were deleted following verbatim transcription. During transcription and subsequent analysis, all personally identifiable information was removed. The interview data were factually anonymized, and results were presented only in aggregated form, making individual identification possible only with disproportionate effort. All data were stored on secure, password-protected systems.

## Results

### Rapid Review Stage 1

**Figure 2:**
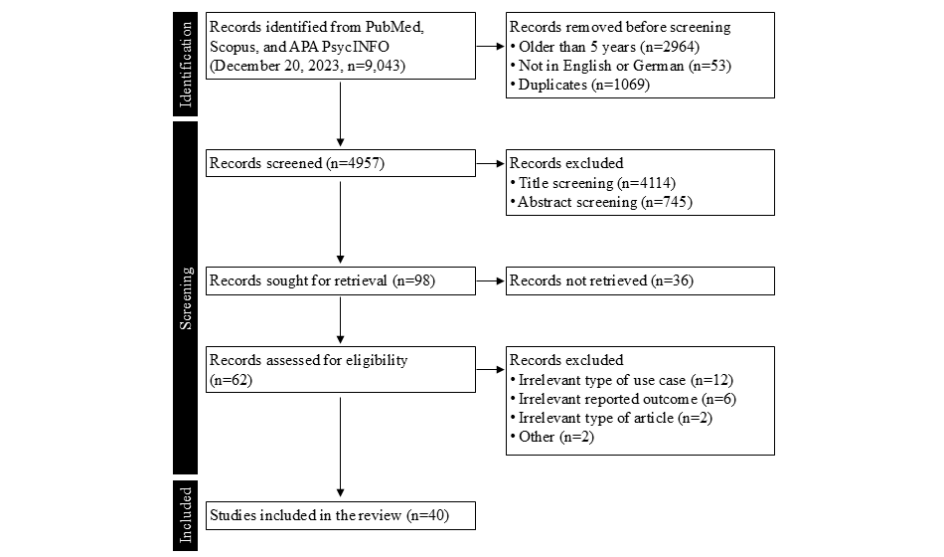
Flowchart of literature screening for rapid review stage 1 following the PRISMA (Preferred Reporting Items for Systematic Reviews and Meta-Analyses) guidelines to identify validated instruments used to assess the postdeployment effects of digital health solutions in outpatient care. Records were retrieved from PubMed, Scopus, and APA PsycINFO on December 20, 2023 (n=9043), filtered by language (English and German) and publication date (2018-2023). A total of 40 studies met the eligibility criteria and were included in the analysis.

Our objective was to identify which instruments are currently used to measure the effects of DHSs. Our query yielded 9043 publications. To focus on the most current publications, we limited our inclusion to articles in English or German and published within the last 5 years, up to 2023. After removing duplicates, 4957 articles remained for screening, which resulted in 98 potentially relevant articles. Of these, 62 articles were accessible and underwent full-text screening, ultimately leading to the inclusion of 40 publications (Figure 2).

In the 40 eligible studies, we identified a total of 126 reported outcomes. We applied the eligibility criteria used during literature screening to these outcomes analogously, resulting in 85 quantitative, nonhealth-related outcomes applied in outpatient settings measuring the postdeployment effects of selected digital use cases. Many of these were based on self-constructed instruments without reporting validity or reliability characteristics. Only 23 were based on instruments that have either been validated or have been used in previous publications [32,63-76].

In total, we identified 18 distinct instruments (Table 1). We assessed the instruments to ensure adherence to our predefined construction criteria, especially for applicability across digital use cases and perspectives. None of the instruments fulfilled both criteria; instead, 3 instruments assessed both the patient’s and HCPs’ perspectives but were use case–specific [63,67,69,72], and 9 instruments were not use case–specific but did not assess different perspectives [32,64-68,70,73,74,76].

**Table 1:** Instruments identified in rapid review stage 1 for evaluating the postdeployment effects of DHSs^a^ in outpatient care, selected based on represented perspectives, use case specificity, and occurrence^b^.

Instrument	Applied to multiple perspectives	Not specific for a DHS use case	Mentions, n	Identified publications
ACES^c^	No	No	1	[74]
CAHPS^d^^,e^	No	Yes	2	[64,66]
Deloitte US health care consumer survey	No	Yes	1	[73]
GMC^f^ Patient Questionnaire	No	Yes	1	[67]
HCCQ^g^	No	Yes	1	[66]
JSPPPE^h^	No	Yes	1	[64]
MISS-21^i^	No	No	1	[76]
OPEQ^e,^^j^	No	Yes	2	[32,65]
PEPPI^k^	No	Yes	1	[74]
STOHFLA^l^	No	Yes	1	[66]
Smartphone Use Score	Yes	No	1	[69]
SUS^m^	Yes	No	1	[69]
TAM^n^	No	Yes	1	[68]
TESS^o^	No	No	1	[67]
TUQ^p^	Yes	No	3	[63,67,72]
TMPQ^q^	No	No	1	[66]
TSUQ^r^	No	No	1	[67]
Trust in Physician Scale	No	No	1	[70]

^a^DHS: digital health solution.

^b^A total of 18 instruments were identified and reviewed for adherence to the predefined criteria; compliance with the following criteria was ensured through the previous selection steps: (1) postdeployment evaluation, (2) non–health related, and (3) application in an outpatient setting; open criteria were checked: applicability across multiple perspectives and digital use cases; and the number of mentions is an additional criterion for further selection.

^c^ACES: Ambulatory Care Experience Survey.

^d^CAHPS: Consumer Assessment of Health Care Providers and Systems.

^e^Instruments that were selected for further analysis.

^f^GMC: General Medical Council.

^g^HCCQ: Health Care Climate Questionnaire.

^h^JSPPPE: Jefferson Scale of Patient Perception of Physician Empathy.

^i^MISS-21: Medical Interview Satisfaction Scale.

^j^OPEQ: Outpatient Experience Questionnaire.

^k^PEPPI: Perceived Efficacy in Patient-Physician Interactions Scale.

^l^STOHFLA: Short test of functional health literacy in adults.

^m^SUS: System Usability Score.

^n^TAM: Technology Acceptance Model.

^o^TESS: Telehealth Satisfaction Scale.

^p^TUQ: Telemedicine Satisfaction and Usefulness Questionnaire.

^q^TMPQ: Telemedicine Perception Questionnaire Score.

^r^TSUQ: Telehealth Usability Questionnaire.

For further instrument development, we hypothesized that adjusting an existing instrument from the patient perspective to the HCP perspective would require fewer changes to the original instrument than making a use case–specific instrument more generalizable.

**Figure 3:**
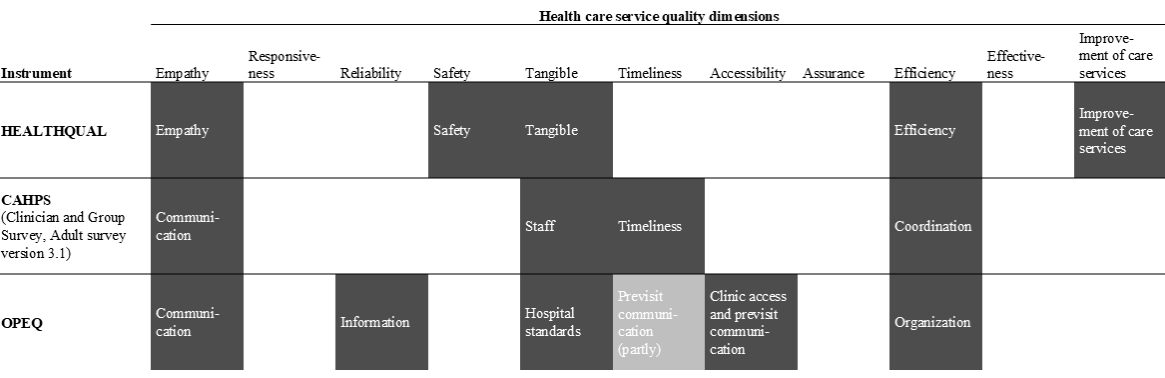
Mapping of instrument dimensions against health care service quality dimensions. Three instruments (Health Care Service Quality [HEALTHQUAL], Consumer Assessment of Health Care Providers and Systems [CAHPS], and Outpatient Experience Questionnaire [OPEQ]) align with common health care service quality dimensions according to Lee [26]. Shaded cells indicate conceptual overlaps between instrument-specific domains and health care service quality dimensions.

To select an instrument, we focused on those that had been applied multiple times, which is the case for the Consumer Assessment of Health Care Providers and Systems (CAHPS) [64,66] and OPEQ [32,65]. We mapped their dimensions against the HCSQ dimensions outlined by Lee [26]. The comparison revealed that both CAHPS and OPEQ cover dimensions similar to those of HEALTHQUAL (Figure 3 [26]). Notably, OPEQ covers a slightly broader range of HCSQ dimensions compared to HEALTHQUAL. Therefore, we selected OPEQ for further use, merged mapping dimensions with HEALTHQUAL, and added accessibility (merging clinic access and previsit communication) and information as additional HCSQ dimensions.

### HCP Interviews

Given that HEALTHQUAL and OPEQ have not been previously used with HCPs, we conducted interviews with HCPs with two objectives: (1) to confirm existing and derive new dimensions for the evaluation of HCSQ from the perspective of HCPs and (2) to understand the importance of each dimension for the overall HCSQ.

**Figure 4:**
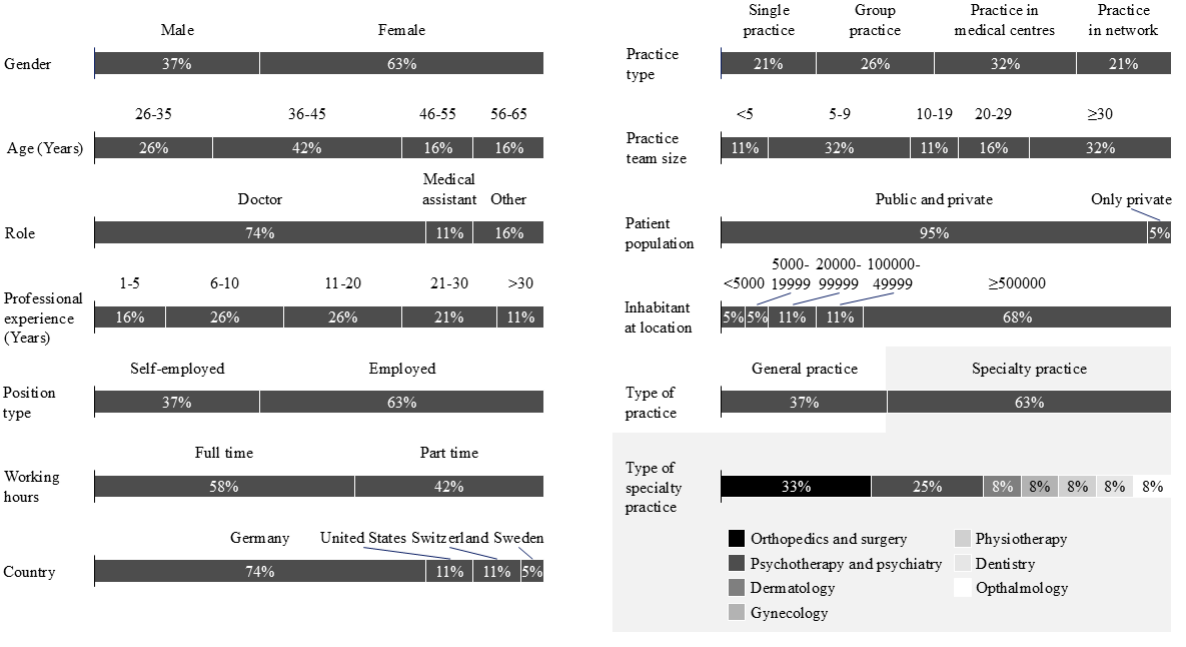
Demographic and professional characteristics of interviewed health care practitioners (n=19). Descriptive statistics of health care practitioners participating in semistructured, qualitative interviews conducted between 2023 and 2025 across Germany, the United States, Switzerland, and Sweden. Variables include gender, age, profession, professional experience, position type, working hours, country, practice type, team size, patient population, size of the location, type of practice, and specialty.

We interviewed 19 HCPs with diverse backgrounds (Figure 4). We derived a total of 11 HCSQ dimensions from the interviews, with a broad consensus for four of them (mentioned by at least 12/19, 60% of interviewees):

1. Accessibility (17/19, 89%); HCPs agreed that being accessible via phone, email, and at the front desk is crucial.
2. Efficient administration (16/19, 16%); HCPs highlighted the importance of efficient administrative processes to keep patient waiting times low.
3. Availability of appointments (13/19, 68%); HCPs pointed out the significance of having appointments available within an acceptable timeframe.
4. Patient interaction (12/19, 63%); HCPs emphasized the importance of spending enough time with patients and maintaining a close physician-patient relationship.

Other less frequently mentioned dimensions included providing information, the atmosphere in the practice, available medical offerings, and improved health conditions. These dimensions match those in HEALTHQUAL and OPEQ. Notably, 4 (21%) out of 19 interviewees explicitly mentioned patient satisfaction as a separate dimension of HCSQ not represented by the dimensions of HEALTHQUAL and OPEQ. The 2 dimensions not represented by the dimensions of HEALTHQUAL and OPEQ were mentioned but with low frequency. Two interviewees mentioned integrated care and the continuous effort of the practice to improve the service.

**Figure 5:**
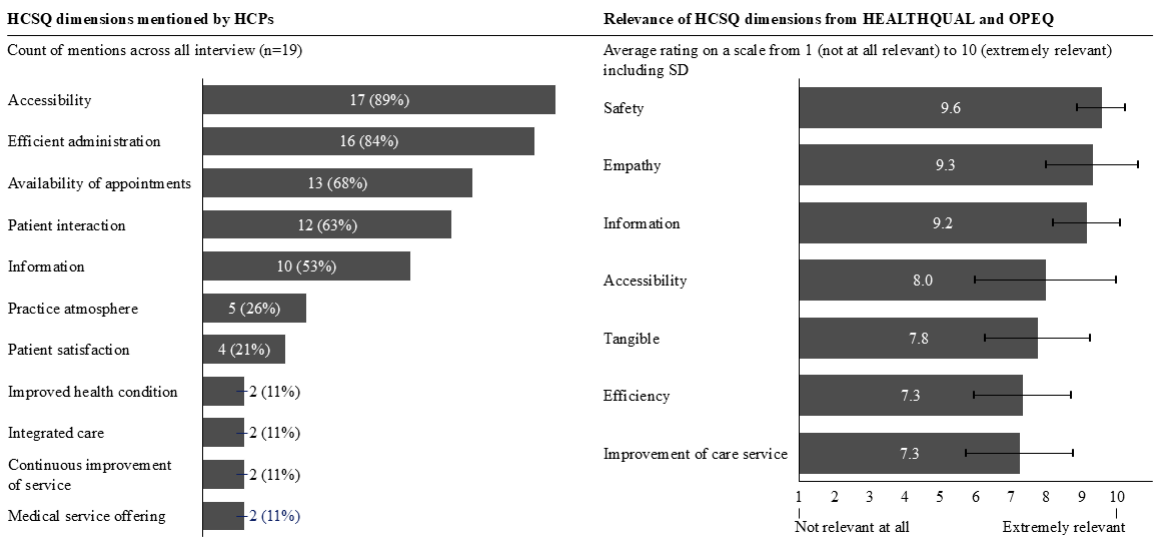
Dimensions of health care service quality (HCSQ) identified and rated by health care practitioners (n=19). Left panel: the frequency with which HCSQ dimensions were mentioned across 19 interviews with outpatient care providers. Right panel: relevance ratings (scale: 1=not at all relevant and 10=extremely relevant) of predefined dimensions from the Health Care Service Quality (HEALTHQUAL) and Outpatient Experience Questionnaire (OPEQ). Bars indicate mean ratings with SD. HCP: health care practitioner.

Furthermore, interviewees rated the relevance of the HEALTHQUAL and OPEQ dimensions (Figure 5). We found that there was a consensus regarding the most relevant HCSQ dimension (safety) but more variation regarding the remaining dimensions. However, all dimensions received a rating of at least 7 on a scale of 10, so we considered all of them as relevant.

These findings confirmed the relevance of the selected HCSQ dimensions for HCPs. However, patient satisfaction was mentioned by >20% (4/19) of interviewees as an additional relevant dimension. Consequently, we conducted another rapid review to identify instruments measuring patient satisfaction in the literature to be added to our questionnaire.

### Rapid Review Stage 2

**Figure 6:**
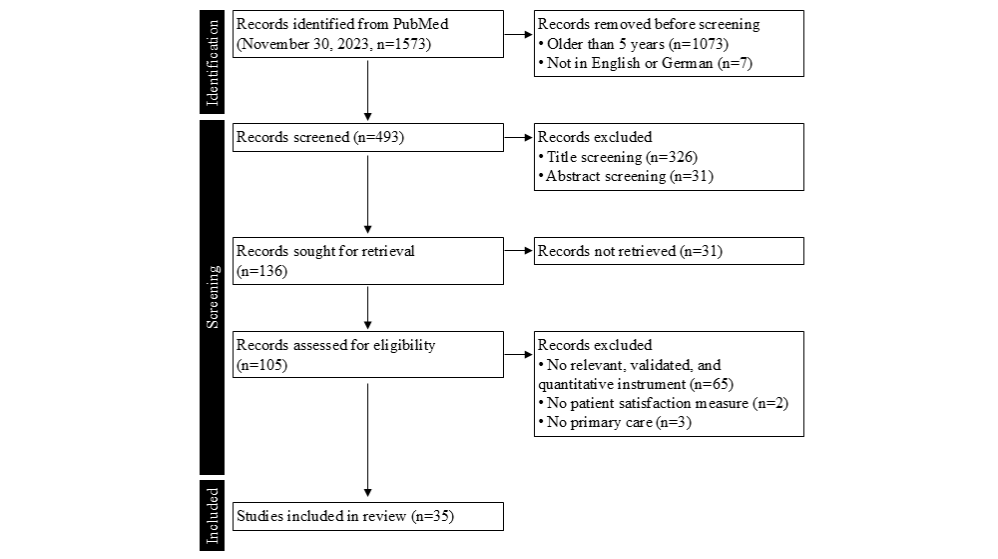
Flowchart of literature screening for rapid review stage 2 following the PRISMA (Preferred Reporting Items for Systematic Reviews and Meta-Analyses) guidelines to identify validated patient satisfaction instruments used in primary health care. Records were retrieved from PubMed on November 30, 2023 (n=1573), filtered by language (English and German) and publication date (2018-2023). A total of 35 studies met the eligibility criteria and were included in the analysis.

The objective was to identify the instruments currently used to measure patient satisfaction. We decided to narrow down the scope of this review further to primary health care settings because the 4 mentions of patient satisfaction were made by HCPs working in primary health care. Our initial search resulted in 1573 publications. To focus on recent research, we included only articles written in English or German that were published within the last 5 years, up to 2023, reducing the dataset to 493 articles. Further screening narrowed the selection to 136 potentially relevant articles. Of these, 105 were accessible and subjected to full-text review, leading to the selection of 35 publications (Figure 6).

In the 35 eligible studies, 29 different instruments related to patient satisfaction were used or mentioned (Tables 2 and 3). To select the most suitable instrument to add to our questionnaire, we evaluated the following criteria: (1) the frequency of use, (2) the internal consistency (ie, Cronbach α), and (3) the feasibility for integration (ie, number of items).

**Table 2:** Instruments identified in rapid review stage 2 for measuring patient satisfaction in primary health care settings, selected based on occurrence^a^.

Instrument (cluster)	Clustered instruments (if applicable)	Applied, n	Mentioned, n	References
PSQ^b,c^	PSQ II, PSQ III, and PSQ-18^d^	6	2	[77-82]
CAHPS^c,e^	Clinician and group version, emergency department version, hospital version, and patient-centered medical home version	6	0	[83-88]
PCAT^c,f^	—^g^	3	1	[89-92]
EUROPEP^c,h^	—	2	2	[90,93-95]
GPAQ^c,i^	GPAQ and GPAQ-R2^j^	2	1	[89,90,96]
AAFP^k^-MGMA^l^ Patient Satisfaction Survey^c^	—	2	0	[97,98]
CQ^m^ Index	—	1	0	[99]
Client Satisfaction Questionnaire	—	1	0	[100]
Tool to Improve Quality of Health Care	—	1	0	[101]
EORTC^n^ Satisfaction with Care Questionnaire	—	1	0	[102]
General Practitioner Patient Survey	—	1	0	[103]
Medical Interview Satisfaction Scale	—	1	0	[104]
Primary Care Consultation Questionnaire	—	1	0	[105]
Paediatric Otolaryngology Telemedicine Satisfaction Survey	—	1	0	[67]
PROMIS^o^	—	1	0	[86]
Patient Satisfaction Survey Questionnaire	—	1	0	[106]
Patient Survey for Quality of Care Scale	—	1	0	[107]
Satisfaction with Stroke Care Questionnaire	—	1	0	[108]
Survey of Healthcare Experiences of Patients	—	1	0	[87]
Telemedicine Satisfaction Questionnaire	—	1	0	[81]
WHO SARA^p^	—	1	0	[101]
Components of Primary Care Index	—	0	2	[89,90]
GMC^q^ Patient Questionnaire	—	0	1	[67]
General Practice Assessment Survey	—	0	1	[90]
General Satisfaction Questionnaire	—	0	1	[108]
SERVQUAL^r^	—	0	1	[79]
TESS^s^	—	0	1	[67]
TSUQ^t^	—	0	1	[67]
TUQ^u^	—	0	1	[67]

^a^A total of 29 instruments were identified in 35 papers; the identified instruments were reviewed in 2 waves; in wave 1, the selection was based on the frequency of use.

^b^PSQ: Patient Satisfaction Questionnaire.

^c^Instruments that were selected for further evaluation.

^d^PSQ-18: Patient Satisfaction Questionnaire-Short Form.

^e^CAHPS: Consumer Assessment of Health Care Providers and Systems.

^f^PCAT: Primary Care Assessment Tool.

^g^Not applicable.

^h^EUROPEP: European Task Force on Patient Evaluations of General Practice Care.

^i^GPAQ: General Practice Assessment Questionnaire.

^j^GPAQ-R2: General Practice Assessment Questionnaire Revised Version 2.

^k^AAFP: American Academy of Family Physicians.

^l^MGMA: Medical Group Management Association.

^m^CQ Index: Consumer Quality Index.

^n^EORTC: European Organization for Research and Treatment of Cancer.

^o^PROMIS: Patient-Reported Outcomes Measurement Information System.

^p^WHO SARA: World Health Organization Service Availability and Readiness Assessment.

^q^GMC: General Medical Council.

^r^SERVQUAL: Service Quality.

^s^TESS: Telehealth Satisfaction Scale.

^t^TSUQ: Telemedicine Satisfaction and Usefulness Questionnaire.

^u^TUQ: Telehealth Usability Questionnaire.

**Table 3:** Comparison of patient satisfaction instruments selected from results of the rapid review stage 2 based on internal consistency and feasibility of integration in future research (approximated through the number of items)^a^.

Instrument (cluster)	Internal consistency (Cronbach α)	Items, n	Additional references
PSQ^b^	0.91^c^	≥18	[34,109]
CAHPS^d^	0.61-0.91^e^	32	[110]
PCAT^f^	0.59-0.94	≥81	[111,112]
EUROPEP^g^	0.81-0.95	23	[113,114]
GPAQ^h^	0.86-0.97^i^	19	[115]
AAFP^j^-MGMA^k^ Patient Satisfaction Survey	>0.7	23	[116]

^a^A total of 29 instruments were identified in 35 papers; the identified instruments were reviewed in 2 waves; in wave 2, selection was based on internal consistency and feasibility for integration.

^b^PSQ: Patient Satisfaction Questionnaire.

^c^PSQ-18. Patient Satisfaction Questionnaire-Short Form.

^d^CAHPS: Consumer Assessment of Health Care Providers and Systems.

^e^Clinician group and patient-centered medical home version.

^f^PCAT: Primary Care Assessment Tool.

^g^EUROPEP: European Task Force on Patient Evaluations of General Practice Care.

^h^GPAQ: General Practice Assessment Questionnaire.

^i^GPAQ-R2: General Practice Assessment Questionnaire Revised Version 2.

^j^AAFP: American Academy of Family Physicians.

^k^MGMA: Medical Group Management Association.

A total of 6 instruments (accounting for different variations or versions of an instrument as 1) were used multiple times. All of them demonstrated overall acceptable Cronbach α values. However, CAHPS and Primary Care Assessment Tool were reported to have Cronbach α values <0.7. The Patient Satisfaction Questionnaires and the General Practice Assessment Questionnaire contained the shortest instruments, with 18 and 19 items, respectively. On the basis of these criteria, we selected the most recent version of the Patient Satisfaction Questionnaires, the PSQ-18, for inclusion in our questionnaire based on its widespread use in the literature, reliability, and feasibility.

### DigiHEALTHQUAL Development

We developed DigiHEALTHQUAL by synthesizing 3 validated instruments—HEALTHQUAL, OPEQ, and PSQ-18—guided by insights from our rapid reviews and interviews in the previous steps. HEALTHQUAL measures HCSQ via 32 items across 5 dimensions (empathy, efficiency, safety, tangible, and degree of improvements of care services), with responses rated on a 5-point scale ranging from “very bad” to “very good” [26]. The OPEQ assesses outpatient experiences through 24 items in 6 dimensions (clinic access, communication, hospital standards, organization, previsit communication, and quality of information), using a 10-point scale with anchoring phrases for each end of the scale [31,32]. The PSQ-18 evaluates patient satisfaction via 18 items across 7 domains (accessibility and convenience, communication, financial aspects, general satisfaction, interpersonal manner, technical quality, and time spent with doctors) on a 5-point Likert-like scale ranging from “strongly agree” to “strongly disagree” [34].

The 3 instruments were compared item by item to remove duplicates and ensure contextual applicability by adapting or removing items as needed (Multimedia Appendix 5). Of the initially longlisted 74 items, 13 were adjusted to better fit the focus of our study, 24 were removed because of redundancy or to account for differences between national health care systems, and 1 new item was added because adjusting the existing question to fit the focus of this study was not feasible (ie, data security considerations).

Subsequently, the remaining dimensions of the 3 original instruments were merged based on content overlap where feasible. The resulting DigiHEALTHQUAL questionnaire consists of 51 items across 8 dimensions: accessibility, efficiency, empathy, general satisfaction, degree of improvements of care services, information, safety, and tangibles.

This current questionnaire draft is designed to use a response scale similar to that of the PSQ-18, a 6-point Likert scale ranging from “completely disagree” to “completely agree,” allowing calculation of a satisfaction score for each subdimension. This scale has proven effective in practice [12]. Consequently, all items were converted accordingly.

## Discussion

### Principal Findings

This study reveals key findings regarding the measurement of the effects of DHSs on HCSQ. First, there is a notable lack of consensus on standardized instruments to measure the effects, especially for nonhealth-related outcomes. We hypothesize that this lack of agreement is a reflection of the novelty and wide variety of available DHSs, which consequently makes it challenging for decision makers to compare the effects of different DHSs across diverse contexts.

In contrast, there is greater agreement on the instruments used to measure patient satisfaction, indicating that these tools are well established and widely accepted. This consensus provides a solid foundation for evaluating patient satisfaction in the context of DHSs, offering a reliable starting point for further research.

Finally, instruments typically used to measure HCSQ have not yet been used to assess the impact of DHSs. This gap underscores the need to adapt existing HCSQ instruments for digital use cases. Interestingly, some of the instruments identified in this study include dimensions relevant to HCSQ. This indicates that some level of assessment of HCSQ elements is already taking place, but it is not being explicitly reported as such.

### Comparison to Prior Work

Previous research has highlighted a lack of consensus on instruments for measuring implementation research outcomes in health care, including DHS deployments, primarily because of insufficient psychometric properties [17-19]. Our study confirms this general observation and provides additional insights specific to DHS implementation research outcome measurement. We found that the lack of standardized instruments is particularly pronounced for nonhealth-related outcomes in DHS implementation studies. Many of these studies used self-constructed instruments that neither referenced previous work nor reported validity or reliability properties. This variability and lack of rigor make it challenging to compare findings across different studies and contexts. Consequently, our findings underscore the need to validate new or existing instruments for nonhealth-related outcomes to enhance the reliability and comparability of DHS implementation studies and generate a consensus within the research community related to the applicable instruments.

In our study, we identified instruments to measure the effects of DHSs and several instruments to measure HCSQ. However, none of the currently used instruments explicitly assess the intersection of these 2 areas: how HCSQ is influenced by DHS deployment. Recognizing this gap, we followed the recommendations of Boateng et al [117] by choosing to adapt and combine existing instruments rather than create a questionnaire de novo*.* This approach also aligns with the experimental practice of modifying established instruments such as SERVQUAL to fit specific health care contexts, although it has not yet been widely applied to DHSs [118]. By following this adaptation strategy, we ensure the relevance and applicability of our instruments while maintaining the validity, reliability, and comparability of the results by leveraging well-established and previously validated instruments.

Our HCP interviews revealed the recommendation to add patient satisfaction as an additional dimension to the set of HCSQ dimensions derived from HEALTHQUAL and OPEQ. This recommendation is anecdotally already supported by our rapid review stage 1, for example, where the CAHPS instrument included a question on overall satisfaction. The literature consistently described that there is a correlation between HCSQ and patient satisfaction or happiness in health care settings [119-121]. Various studies identify factors influencing patient satisfaction. For example, Duc Thanh et al [120] identified key factors affecting patient satisfaction, including satisfaction with facilities, service provision, information transparency and administrative procedures, accessibility, and staff interaction and communication. Similarly, Ferreira et al [119] highlighted factors such as waiting times, information provided, cleanliness, communication with patients, and doctors’ characteristics. These factors affecting patient satisfaction align closely with the HCSQ dimensions outlined by Lee [26], underscoring the interconnectedness of the metrics patient satisfaction and HCSQ and their collective impact on health care evaluation—supporting the inclusion of patient satisfaction in DigiHEALTHQUAL.

### Strengths and Limitations

By leveraging existing, validated instruments rather than creating new ones, we ensure comparability with previous studies. This approach not only strengthens the validity and reliability of DigiHEALTHQUAL but also facilitates meaningful comparisons and meta-analyses for future research. In addition, our study aimed to add the HCP perspective to patient-focused instruments, providing a holistic view of HCSQ. This comprehensive approach ensures that the nuances and needs of both key stakeholder groups are considered in future research, enhancing the overall applicability and relevance of DigiHEALTHQUAL. Moreover, our approach aligns with the best practices for developing and validating scales for health research, as described by Boateng et al [117], who describe 9 steps across 3 phases for the development of rigorous scales. Following their work, we completed the first phase, “item development,” comprising the 2 steps “identification of domain and item generation” and “content validity” in this study [117].

To enable broad application, we excluded dimensions from the original instruments, for example, items related to financial aspects, such as “appropriate costs” or “reasonable medical expenses,” because of varying reimbursement across national health care systems. This may result in a lack of insights into selected aspects of HCSQ. Furthermore, the interviews informing our questionnaire design were conducted exclusively with HCPs from high-income countries, which may limit the applicability of the resulting questionnaire to countries with different health care systems. However, the interviews only informed the decision to keep the HCSQ dimensions derived from the validated instruments HEALTHQUAL and OPEQ and to add patient satisfaction as an additional dimension—also derived from a validated instrument used in international studies. Therefore, we estimate that the geographic bias of the interviewees is negligible at this stage of our research. It is important to note that our resulting country-unspecific instrument may have limitations in practice, where national regulatory frameworks influence the adoption of DHSs and data use [15,122].

Generalization issues also arise, as some selected digital use cases may not impact all included HCSQ dimensions. For example, we theorize that online appointment booking platforms are rather unlikely to affect health outcomes. Furthermore, nonprioritized DHSs may require other evaluation criteria. For instance, we hypothesize that the effects of Digital Health Applications will materialize mainly in health outcomes rather than HCSQ and, therefore, necessitate specific, targeted assessment tools. This underscores the need for researchers to balance between using broadly applicable instruments that facilitate comparison across studies and specific instruments that provide detailed insights.

Within the broader context of health care, it is important to note the following: merely assessing the effects of DHSs is insufficient to accelerate their adoption. Comprehensive implementation strategies are necessary to facilitate widespread use.

Moreover, while scaling the digitalization of health care can improve health care delivery, it may also reinforce or exacerbate existing disparities. Health care access and quality are already unevenly distributed, and DHSs risk exacerbating these existing inequalities [123].

On the one hand, digital technologies can help mitigate disparities, for example, by improving access in underserved areas or personalizing care. On the other hand, they can also create new barriers for populations with limited digital literacy, lower socioeconomic status, or restricted access to technology. Studies show that the use of digital health tools correlates with sociodemographic factors such as age; income; education; and, critically, health and digital literacy [124-126].

This introduces 2 challenges. First, in the context of validating DigiHEALTHQUAL, it is important that diverse user groups are appropriately represented. Digitally literate individuals are easier to recruit through online channels, whereas individuals with limited digital access or skills may be systematically underrepresented, leading to bias in the findings. Second, beyond the scope of our work, there is a need for comprehensive planning that ensures equity of access. DHSs must be designed and implemented in a way that safeguards against creating additional barriers and addresses common user concerns such as data privacy, usability, and accessibility.

### Practical Implication and Further Research

The implications for further research based on our study are threefold.

#### Scale Development and Evaluation

Following the best practices for developing and validating scales as outlined by Boateng et al [117], it is necessary to complete the next phases of scale development and evaluation to ensure the validity and reliability of the DigiHEALTHQUAL questionnaire. This involves conducting validation studies with patients to test the resulting questionnaire and subsequently performing validation studies with HCPs to adjust DigiHEALTHQUAL to reflect their perspectives as well. The authors have already initiated this process by conducting preliminary face validity testing with 10 representatives from each target group—patients and HCPs. On the basis of their feedback, minor adjustments were made to improve clarity and contextual relevance. Full-scale quantitative validation studies are currently in preparation, aiming to collect statistically adequate samples for each group. These studies are expected to conclude by the end of 2025 and will include psychometric analyses to support refinement, including item reduction and dimensional validation. To ensure methodological rigor, the planned validation will apply exploratory and confirmatory factor analysis to assess the dimensional structure, internal consistency testing (eg, Cronbach α), and construct validity evaluation. Each quantitative study will aim to include a minimum of 200 participants per target group (patients and HCPs).

#### Data Collection From DHS Deployment Studies

Collecting data from DHS deployment studies will enable decision makers to retrospectively assess whether the impact they aimed for has been realized after deployment. This data collection is crucial for understanding the real-world effects of DHSs and refining the evaluation tools based on practical insights.

#### Meta-Analysis of Postdeployment Studies

Conducting meta-analyses across postdeployment studies using DigiHEALTHQUAL will allow decision makers to prospectively consider the effects of DHSs and make informed decisions before deployment. This is especially important in resource-constrained environments, such as those commonly encountered in health care, where efficient allocation of resources is critical.

To optimize the impact of this research, we suggest initially focusing on use cases with the highest current implementation rates. Our prioritization of 4 use cases can serve as an initial starting point. In addition, there should be a strong emphasis on the outpatient setting, as digital transformation significantly lags behind in outpatient care compared to inpatient care [9,10]. A robust evaluation tool would be particularly valuable in outpatient settings, where clear and actionable guidance is critical for choosing among the many available DHSs that cater to patients with diverse health needs. In these settings, decision makers are often physicians who must juggle multiple roles. We hypothesize that these physicians have limited capacity to make business decisions or compare DHS offerings, given the workforce shortages and growing demand for health care services. Therefore, the decision makers need a robust evaluation framework even more than larger health care organizations with dedicated functions in place.

### Conclusions

Our study underscores the importance of developing and validating instruments to measure the effects of DHS deployment on HCSQ in real-life settings. By adapting and combining validated instruments, we provide a robust framework for assessing HCSQ, extending their application to the new and less-explored context of digital health.

Currently, decision makers face significant challenges in comparing outcomes from digital health implementation research because of the lack of consensus on evaluation tools, the use of many different instruments tailored to specific digital use cases, and the variance in the quality of the applied instruments. Although existing tools for HCSQ have not yet been used to assess the effects of DHSs, instruments for digital tools often evaluate relevant HCSQ factors but lack broad applicability.

We propose a comprehensive set of dimensions to measure HCSQ based on the validated instruments HEALTHQUAL, OPEQ, and PSQ-18. This set can be used by decision makers and practitioners to assess ongoing or completed DHS deployments. However, future research is essential to validate the reliability and validity of this scale further and to collect a robust database that can support pre-deployment decision-making in the future.

In summary, DigiHEALTHQUAL aims to fill the gap in current digital health evaluation practices, providing a standardized approach that enhances the comparability of research outcomes and ultimately supports better decision-making in the implementation of DHSs.

## Appendix

Multimedia Appendix 1 PRISMA-ScR checklist—rapid review stage 1.

Multimedia Appendix 2 PRISMA-ScR checklist—rapid review stage 2.

Multimedia Appendix 3 COREQ checklist—health care practitioner interviews.

Multimedia Appendix 4 Translated semistructured interview guide.

Multimedia Appendix 5 Development of the Digital Healthcare Service Quality Questionnaire.

## Abbreviations

CAHPS: Consumer Assessment of Health Care Providers and Systems
COREQ: Consolidated Criteria for Reporting Qualitative Research
DHS: digital health solution
DigiHEALTHQUAL: Digital Healthcare Service Quality
HCP: health care practitioner
HCSQ: health care service quality
HEALTHQUAL: Health Care Service Quality
OPEQ: Outpatient Experience Questionnaire
PRISMA: Preferred Reporting Items for Systematic Reviews and Meta-Analyses
PRISMA-RR: Preferred Reporting Items for Systematic Reviews and Meta-Analyses Extension for Rapid Reviews
PRISMA-ScR: Preferred Reporting Items for Systematic Reviews and Meta-Analyses Extension for Scoping Reviews
PSQ-18: Patient Satisfaction Questionnaire-Short Form
SERVQUAL: Service Quality

## Data Availability

The datasets generated or analyzed during this study are available from the corresponding author on reasonable request.
