# Supplementary material for: Service Quality Assessment of Digital Health Solutions in Outpatient Care: Qualitative Item Repository Development Study
Source: JMIR Form Res. 2025 Jul 24;9:e68276. doi: 10.2196/68276 (PMC12332462; doi:10.2196/68276)
Supplement: Multimedia Appendix 1 [file formative_v9i1e68276_app1.pdf]

Multimedia Appendix 1: PRISMA-ScR Checklist – Rapid Review Stage 1.

SECTION	ITEM	PRISMA-ScR CHECKLIST ITEM	REPORTED IN SECTION
**TITLE**			
Title	1	Identify the report as a scoping review.	n/a
**ABSTRACT**			
Structured summary	2	Provide a structured summary that includes (as applicable): background, objectives, eligibility criteria, sources of evidence, charting methods, results, and conclusions that relate to the review questions and objectives.	**Background & Objective:** The integration of digital health solutions into healthcare systems holds significant potential for improving service delivery and health outcomes. However, adoption is slow, especially in outpatient care, due to implementation barriers, such as uncertain effectiveness and high costs. Additionally, the complexity of the digital health ecosystem and variability in the scope and quality of evaluation instruments complicate decision-making. This study aims to identify, adapt, or develop a comprehensive instrument to evaluate the effects of digital health solutions. The first stage focuses on identifying instruments currently used to assess post-deployment effects of digital tools.<br>**Method:** We searched three databases and filtered for articles in English or German published within the last five years (up to 2023). After deduplication and manual title screening, we used ASReview LAB, an AI-assisted tool, for abstract screening. Inclusion criteria were based on article type, reported outcomes, and use cases. We synthesized data by extracting details on findings and applied instruments.<br>**Results:** From 9,043 initially identified studies, 40 were included. Most studies were observational (30/40), conducted in a single country (38/40), focused on video consultations (35/40), included only the patient perspective (38/40), and exclusively took place in outpatient settings (35/40). We identified 126 outcomes, 90 of which were non-health related. Of these, 28% cited validated instruments, covering 15 studies and 20 instruments. None met all of our five criteria for an evaluation instrument.<br>**Discussion:** Many studies investigate the effects of digital tools in healthcare but rarely across multiple setups and, especially for non-health related outcomes, with varying validity and reliability. While healthcare service quality has not been explicitly measured as an

SECTION	ITEM	PRISMA-ScR CHECKLIST ITEM	REPORTED IN SECTION
			outcome, relevant dimensions were included in the most reliable instruments which fulfilled most of our selection criteria. The Outpatient Experience Questionnaire had the broadest coverage of healthcare service quality and was selected for further research.
**INTRODUCTION**			
Rationale	3	Describe the rationale for the review in the context of what is already known. Explain why the review questions/objectives lend themselves to a scoping review approach.	The benefits of digitalization in healthcare are well-documented, yet adoption remains slow compared to other sectors. One of the key barriers to implementation is the need for decision-makers to weigh the effectiveness of digital health solutions against their costs. This task is becoming increasingly challenging due to the growing complexity of the digital health landscape and the limited comparability of effectiveness assessments. Currently, there is a lack of consensus on the instruments used to measure the effects of digital health solutions, with existing instruments varying widely in reliability and scope. This inconsistency complicates the ability of decision-makers to make informed choices. Given these challenges, our review aims to identify, adapt, or develop a comprehensive instrument to evaluate the effects of digital health solutions focusing on outpatient settings. The scoping review approach is particularly suited to this objective as it allows for a broad examination of the available evidence, mapping of the key concepts, types of evidence, and gaps in research.
Objectives	4	Provide an explicit statement of the questions and objectives being addressed with reference to their key elements (e.g., population or participants, concepts, and context) or other relevant key elements used to conceptualize the review questions and/or objectives.	Our objective was to identify which instruments are currently utilized to measure the effects of digital health solutions following our five selection criteria as described in '*2.2 Focus of This Study*'.
**METHODS**			
Protocol and registration	5	Indicate whether a review protocol exists; state if and where it can be accessed (e.g., a Web address); and if available, provide registration information, including the registration number.	As this review was part of a more comprehensive study approach, we did not prepare, register, and publish a review protocol.
Eligibility criteria	6	Specify characteristics of the sources of evidence used as eligibility criteria (e.g., years considered, language, and publication status), and provide a rationale.	see *'Rapid Review Stage 1'*
Information sources	7	Describe all information sources in the search (e.g., databases with dates of coverage and contact with authors	see *'Rapid Review Stage 1'*

SECTION	ITEM	PRISMA-ScR CHECKLIST ITEM	REPORTED IN SECTION
		to identify additional sources), as well as the date the most recent search was executed.	
Search	8	Present the full electronic search strategy for at least 1 database, including any limits used, such that it could be repeated.	We conducted our search across three databases: PubMed, Scopus, and APA PsycInfo. Our search utilized a structured string comprising four conceptual blocks: "Digital health", "Implementation", "Effects", and "Assessment". These blocks were connected using the Boolean operator "AND", with multiple search terms within each block linked by the Boolean operator "OR". For PubMed, MeSH terms specific to digital health were added to refine the search further. The search terms were adapted from recent reviews within this field.<br><br>PubMed: (("Connected health"[Title/Abstract]) OR ("Digital health"[Title/Abstract]) OR ("Digital medicine"[Title/Abstract]) OR ("Digital therapeutics"[Title/Abstract]) OR ("Digital therapy"[Title/Abstract]) OR ("e health"[Title/Abstract]) OR ("electronic care"[Title/Abstract]) OR ("electronic health"[Title/Abstract]) OR ("m health"[Title/Abstract]) OR ("Mobile care"[Title/Abstract]) OR ("Mobile health"[Title/Abstract]) OR ("tele care"[Title/Abstract]) OR ("tele health"[Title/Abstract]) OR ("tele healthcare"[Title/Abstract]) OR ("tele medicine"[Title/Abstract]) OR ("Virtual care"[Title/Abstract]) OR ("wireless health"[Title/Abstract]) OR (eHealth[Title/Abstract]) OR (e-health[Title/Abstract]) OR (mhealth[Title/Abstract]) OR (m-health[Title/Abstract]) OR (telehealth[Title/Abstract]) OR (tele-health[Title/Abstract]) OR (telehealthcare[Title/Abstract]) OR (tele-healthcare[Title/Abstract]) OR (Telemedicine[Title/Abstract]) OR (Tele-medicine[Title/Abstract])) AND ((Adopt*[Title/Abstract]) OR (Implement*[Title/Abstract]) OR (Intervention*[Title/Abstract]) OR (Introduction*[Title/Abstract]) OR (disseminat*[Title/Abstract]) OR (incorporat*[Title/Abstract]) OR (institutionalisation[Title/Abstract]) OR (institutionalization[Title/Abstract]) OR (Integrat*[Title/Abstract]) OR (penetrat*[Title/Abstract]) OR (uptake[Title/Abstract]) OR (utilisation[Title/Abstract]) OR (utilization[Title/Abstract])) AND((implication*[Title/Abstract]) OR (impact*[Title/Abstract]) OR (effect*[Title/Abstract]) OR (outcome*[Title/Abstract]))

SECTION	ITEM	PRISMA-ScR CHECKLIST ITEM	REPORTED IN SECTION
			AND((scale*[Title/Abstract]) OR ("technology evaluation"[Title/Abstract]) OR ("technology assessment"[Title/Abstract]))<br>AND (((telemedicine[MeSH Terms]) OR (medical informatics[MeSH Terms]) OR (mobile applications[MeSH Terms]))<br>AND (delivery of health care[MeSH Terms]))<br><br>Scopus: TITLE-ABS-KEY((("Connected health") OR ("Digital health") OR ("Digital medicine") OR ("Digital therapeutics") OR ("Digital therapy") OR ("e health") OR ("electronic care") OR ("electronic health") OR ("m health") OR ("Mobile care") OR ("Mobile health") OR ("tele care") OR ("tele health") OR ("tele healthcare") OR ("tele medicine") OR ("Virtual care") OR ("wireless health") OR (eHealth) OR (e-health) OR (mhealth) OR (m-health) OR (telehealth) OR (tele-health) OR (telehealthcare) OR (tele-healthcare) OR (Telemedicine) OR (Tele-medicine))<br>AND ((Adopt*) OR (Implement*) OR (Intervention*) OR (Introduction*) OR (disseminat*) OR (incorporat*) OR (institutionalisation) OR (institutionalization) OR (Integrat*) OR (penetrat*) OR (uptake) OR (utilisation) OR (utilization))<br>AND((implication*) OR (impact*) OR (effect*) OR (outcome*))<br>AND((scale*) OR ("technology evaluation") OR ("technology assessment")))<br><br>APA PsycInfo: TI ((("Connected health") OR ("Digital health") OR ("Digital medicine") OR ("Digital therapeutics") OR ("Digital therapy") OR ("e health") OR ("electronic care") OR ("electronic health") OR ("m health") OR ("Mobile care") OR ("Mobile health") OR ("tele care") OR ("tele health") OR ("tele healthcare") OR ("tele medicine") OR ("Virtual care") OR ("wireless health") OR (eHealth) OR (e-health) OR (mhealth) OR (m-health) OR (telehealth) OR (tele-health) OR (telehealthcare) OR (tele-healthcare) OR (Telemedicine) OR (Tele-medicine))<br>AND ((Adopt*) OR (Implement*) OR (Intervention*) OR (Introduction*) OR (disseminat*) OR (incorporat*) OR (institutionalisation) OR (institutionalization) OR (Integrat*) OR (penetrat*) OR (uptake) OR (utilisation) OR (utilization))<br>AND((implication*) OR (impact*) OR (effect*) OR (outcome*))

SECTION	ITEM	PRISMA-ScR CHECKLIST ITEM	REPORTED IN SECTION
			AND((scale*) OR ("technology evaluation") OR ("technology assessment")))<br>OR AB ((("Connected health") OR ("Digital health") OR ("Digital medicine") OR ("Digital therapeutics") OR ("Digital therapy") OR ("e health") OR ("electronic care") OR ("electronic health") OR ("m health") OR ("Mobile care") OR ("Mobile health") OR ("tele care") OR ("tele health") OR ("tele healthcare") OR ("tele medicine") OR ("Virtual care") OR ("wireless health") OR (eHealth) OR (e-health) OR (mhealth) OR (m-health) OR (telehealth) OR (tele-health) OR (telehealthcare) OR (tele-healthcare) OR (Telemedicine) OR (Tele-medicine))<br>AND ((Adopt*) OR (Implement*) OR (Intervention*) OR (Introduction*) OR (disseminat*) OR (incorporat*) OR (institutionalisation) OR (institutionalization) OR (Integrat*) OR (penetrat*) OR (uptake) OR (utilisation) OR (utilization))<br>AND((implication*) OR (impact*) OR (effect*) OR (outcome*))<br>AND((scale*) OR ("technology evaluation") OR ("technology assessment")))
Selection of sources of evidence	9	State the process for selecting sources of evidence (i.e., screening and eligibility) included in the scoping review.	see *'Rapid Review Stage 1'*
Data charting process	10	Describe the methods of charting data from the included sources of evidence (e.g., calibrated forms or forms that have been tested by the team before their use, and whether data charting was done independently or in duplicate) and any processes for obtaining and confirming data from investigators.	We developed a data table sheet listing all selection criteria and relevant data fields for later data extraction from included studies. DR extracted the data accordingly. Data were extracted as reported in the included studies, compared afterwards to harmonize wording of data entries across studies and categorize the data points.
Data items	11	List and define all variables for which data were sought and any assumptions and simplifications made.	We collected data from each eligible report on (1) the report itself (including title, authors, journal, publication date, search database, DOI), (2) the study setting (including type of study, digital use cases, type of healthcare organization, therapeutic area, type/number of included healthcare practitioners, type/number of included patients, name/number of included countries), and (3) the reported outcomes and applied instruments (including reported outcomes, type of outcome, applied instruments, source of applied instruments, subdimensions of applied instruments.

SECTION	ITEM	PRISMA-ScR CHECKLIST ITEM	REPORTED IN SECTION
Critical appraisal of individual sources of evidence	12	If done, provide a rationale for conducting a critical appraisal of included sources of evidence; describe the methods used and how this information was used in any data synthesis (if appropriate).	We did not conduct a critical appraisal of included studies as we aimed to identify the instruments currently applied to measure the effects of digital health solutions and planned to validate the results of this review in qualitative healthcare practitioner interviews, ensuring relevance and completeness of our results.
Synthesis of results	13	Describe the methods of handling and summarizing the data that were charted.	see *'Rapid Review Stage 1'*
**RESULTS**			
Selection of sources of evidence	14	Give numbers of sources of evidence screened, assessed for eligibility, and included in the review, with reasons for exclusions at each stage, ideally using a flow diagram.	see *'Rapid Review Stage 1'*
Characteristics of sources of evidence	15	For each source of evidence, present characteristics for which data were charted and provide the citations.	The detailed characteristics of included studies and the extracted data mentioned above can be obtained from the corresponding author upon request and is not presented in detail.
Critical appraisal within sources of evidence	16	If done, present data on critical appraisal of included sources of evidence (see item 12).	n/a
Results of individual sources of evidence	17	For each included source of evidence, present the relevant data that were charted that relate to the review questions and objectives.	see *'Rapid Review Stage 1'*
Synthesis of results	18	Summarize and/or present the charting results as they relate to the review questions and objectives.	see *'Rapid Review Stage 1'*
**DISCUSSION**			
Summary of evidence	19	Summarize the main results (including an overview of concepts, themes, and types of evidence available), link to the review questions and objectives, and consider the relevance to key groups.	see *'Rapid Review Stage 1'* and *'Principal results'*
Limitations	20	Discuss the limitations of the scoping review process.	Firstly, we applied language filters, limiting our search to articles published in German or English. This exclusion of articles in other languages might have led to the exclusion of relevant studies, introducing a potential bias. Secondly, we limited our search to articles published within the last five years up to 2023. While this ensures the inclusion of the most recent research, particularly in the evolving field of digital health, it also means that older, potentially relevant studies were not considered. Thirdly, we limited our search to three databases: PubMed, Scopus, and PsycInfo. Although these databases cover a wide range of relevant literature, there is a possibility that studies indexed in

SECTION	ITEM	PRISMA-ScR CHECKLIST ITEM	REPORTED IN SECTION
			other databases were missed. Additionally, the inclusion/exclusion criteria, which we used to select studies, i.e. considering the type of publication, type of reported outcome, and type of use case, might have excluded studies with relevant insights from the broader context of digital health solutions. Lastly, the entire process of search, data extraction, and synthesis was conducted by only one author (DR). This lack of validation by a second reviewer could have introduced biases or errors in the study selection and data extraction processes.
Conclusions	21	Provide a general interpretation of the results with respect to the review questions and objectives, as well as potential implications and/or next steps.	Our review suggests that there is already substantial research investigating the effects of digital health solutions in healthcare. However, these studies are rarely conducted across multiple settings and often focus on single use cases or perspectives. Notably, for non-health related outcomes, there is a lack of consensus on instruments, with significant variability in the tools used and their reliability and validity. Our study hypothesized that healthcare service quality might be an outcome applicable across multiple settings. This hypothesis is supported by our findings, as the most reliable and frequently used instruments, which met most of our criteria, contain dimensions that overlap with those of healthcare service quality. Although these instruments do not explicitly investigate healthcare service quality, their components align closely with its dimensions. The Outpatient Experience Questionnaire demonstrated the broadest coverage of healthcare service quality dimensions and was selected for further research. To validate and refine our findings, we plan to conduct interviews with healthcare practitioners to ensure the representation of the healthcare practitioners' perspective, and the relevance and applicability of the Outpatient Experience Questionnaire in assessing the impact of digital health solutions.
**FUNDING**			
Funding	22	Describe sources of funding for the included sources of evidence, as well as sources of funding for the scoping review. Describe the role of the funders of the scoping review.	As this research is part of a doctoral thesis at Witten/Herdecke University, it has not received any funding.
