# Supplementary material for: Service Quality Assessment of Digital Health Solutions in Outpatient Care: Qualitative Item Repository Development Study
Source: JMIR Form Res. 2025 Jul 24;9:e68276. doi: 10.2196/68276 (PMC12332462; doi:10.2196/68276)
Supplement: Multimedia Appendix 2 [file formative_v9i1e68276_app2.pdf]

Multimedia Appendix 2: PRISMA-ScR Checklist – Rapid Review Stage 2.

SECTION	ITEM	PRISMA-ScR CHECKLIST ITEM	REPORTED IN SECTION
**TITLE**			
Title	1	Identify the report as a scoping review.	n/a
**ABSTRACT**			
Structured summary	2	Provide a structured summary that includes (as applicable): background, objectives, eligibility criteria, sources of evidence, charting methods, results, and conclusions that relate to the review questions and objectives.	**Background & Objective**: We compiled a list of healthcare service quality dimensions based on previous literature research with the goal to identify, adapt, or develop a questionnaire to comparably measure the effects of digital health solutions across various healthcare settings. These dimensions were derived from patient-focused studies. Therefore we validated them through interviews with healthcare practitioners, who confirmed their relevance and highlighted patient satisfaction as an additional important dimension. The objective of this second review is to identify instruments currently used to measure patient satisfaction for integration into the final questionnaire.<br>**Method**: We conducted a search in one database, filtering for articles in English or German published within the last five years (up to 2023). Screening was performed using inclusion criteria based on article type, reported outcomes, reported healthcare setting, and reported instruments. Data were synthesized by extracting details on findings and applied instruments.<br>**Results**: From 1,573 initially identified studies, 35 were included. Most studies were conducted in the USA, China, or Saudi Arabia (14/35) and involved smaller cohorts of fewer than 1,000 participants (20/35) in primary care clinics (22/35). We identified 29 different instruments measuring patient satisfaction and evaluated them based on frequency of use, reliability, and feasibility for integration into our final questionnaire.<br>**Discussion**: Many studies investigate patient satisfaction across various settings. However, there is a consensus regarding the applied instruments. Based on our selection criteria, we selected the Patient Satisfaction Questionnaire-Short Form for our study.
**INTRODUCTION**			

SECTION	ITEM	PRISMA-ScR CHECKLIST ITEM	REPORTED IN SECTION
Rationale	3	Describe the rationale for the review in the context of what is already known. Explain why the review questions/objectives lend themselves to a scoping review approach.	The integration of digital health solutions into healthcare systems holds significant potential for improving service delivery and health outcomes. However, adoption remains slow, particularly in outpatient care. Decision-makers must evaluate multiple factors, such as effectiveness and costs, to select and implement digital health solutions. The increasing complexity of the digital health ecosystem and the variability in the scope and quality of reported outcomes complicate this decision-making process. Our study aims to identify, adapt, or develop a comprehensive instrument to evaluate the effects of digital health solutions comparably across settings and perspectives. In a previous step, we identified instruments currently used to assess the effects of digital health solutions. We found that the most used and reliable instruments had overlap with healthcare service quality subdimensions. We selected the Outpatient Experience Questionnaire and added its subdimensions to the list of healthcare service quality dimensions from the established HEALTHQUAL instrument. These dimensions were validated through interviews with healthcare practitioners, who confirmed their relevance and highlighted patient satisfaction as an additional important dimension.
Objectives	4	Provide an explicit statement of the questions and objectives being addressed with reference to their key elements (e.g., population or participants, concepts, and context) or other relevant key elements used to conceptualize the review questions and/or objectives.	Our objective was to identify which instruments are currently utilized to measure patient satisfaction in primary healthcare.
**METHODS**			
Protocol and registration	5	Indicate whether a review protocol exists; state if and where it can be accessed (e.g., a Web address); and if available, provide registration information, including the registration number.	As this review was part of a more comprehensive study approach, we did not prepare, register, and publish a review protocol.
Eligibility criteria	6	Specify characteristics of the sources of evidence used as eligibility criteria (e.g., years considered, language, and publication status), and provide a rationale.	see *'Rapid Review Stage 2'*
Information sources	7	Describe all information sources in the search (e.g., databases with dates of coverage and contact with authors to identify additional sources), as well as the date the most recent search was executed.	see *'Rapid Review Stage 2'*

SECTION	ITEM	PRISMA-ScR CHECKLIST ITEM	REPORTED IN SECTION
Search	8	Present the full electronic search strategy for at least 1 database, including any limits used, such that it could be repeated.	We conducted our search at the database PubMed. Our search utilized a structured string comprising three conceptual blocks: "Patient satisfaction", "Assessment", and "Primary healthcare". These blocks were connected using the Boolean operator "AND", with multiple search terms within each block linked by the Boolean operator "OR": (("Patient satisfaction"[Title/Abstract])) AND ((evaluation*[Title/Abstract]) OR (assessment*[Title/Abstract]) OR (survey*[Title/Abstract]) OR (instrument*[Title/Abstract]) OR (tool*[Title/Abstract]) OR (questionnaire*[Title/Abstract])) AND (("primary healthcare"[Title/Abstract]) OR ("primary care"[Title/Abstract]) OR ("primary health care"[Title/Abstract]))
Selection of sources of evidence	9	State the process for selecting sources of evidence (i.e., screening and eligibility) included in the scoping review.	see *'Rapid Review Stage 2'*
Data charting process	10	Describe the methods of charting data from the included sources of evidence (e.g., calibrated forms or forms that have been tested by the team before their use, and whether data charting was done independently or in duplicate) and any processes for obtaining and confirming data from investigators.	We developed a data table sheet listing all selection criteria and relevant data fields for later data extraction from included studies. DR extracted the data accordingly. Data were extracted as reported in the included studies, compared afterwards to harmonize wording of data entries across studies and categorize the data points.
Data items	11	List and define all variables for which data were sought and any assumptions and simplifications made.	We collected data from each eligible report on (1) the report itself (including title, authors, journal, publication date, DOI), (2) the study setting (including type of primary care setting, therapeutic area, type/number of included patients, name/number of included countries), and (3) the reported outcomes and applied instruments (including reported outcomes related to patient satisfaction, applied instruments, source of applied instruments, number/name of subdimensions of applied instruments, number of items.
Critical appraisal of individual sources of evidence	12	If done, provide a rationale for conducting a critical appraisal of included sources of evidence; describe the methods used and how this information was used in any data synthesis (if appropriate).	We did not conduct a critical appraisal of included studies as we aimed to identify the instruments currently applied to measure patient satisfaction based on previous validation in qualitative healthcare practitioner interviews.
Synthesis of results	13	Describe the methods of handling and summarizing the data that were charted.	see *'Rapid Review Stage 2'*
**RESULTS**			

SECTION	ITEM	PRISMA-ScR CHECKLIST ITEM	REPORTED IN SECTION
Selection of sources of evidence	14	Give numbers of sources of evidence screened, assessed for eligibility, and included in the review, with reasons for exclusions at each stage, ideally using a flow diagram.	see *'Rapid Review Stage 1'*
Characteristics of sources of evidence	15	For each source of evidence, present characteristics for which data were charted and provide the citations.	The detailed characteristics of included studies and the extracted data mentioned above can be obtained from the corresponding author upon request and is not presented in detail.
Critical appraisal within sources of evidence	16	If done, present data on critical appraisal of included sources of evidence (see item 12).	n/a
Results of individual sources of evidence	17	For each included source of evidence, present the relevant data that were charted that relate to the review questions and objectives.	see *'Rapid Review Stage 2'*
Synthesis of results	18	Summarize and/or present the charting results as they relate to the review questions and objectives.	see *'Rapid Review Stage 2'*
**DISCUSSION**			
Summary of evidence	19	Summarize the main results (including an overview of concepts, themes, and types of evidence available), link to the review questions and objectives, and consider the relevance to key groups.	see *'Rapid Review Stage 2'* and *'Principal results'*
Limitations	20	Discuss the limitations of the scoping review process.	Firstly, we applied language filters, limiting our search to articles published in German or English. This exclusion of articles in other languages might have led to the exclusion of relevant studies, introducing a potential bias. Secondly, we limited our search to articles published within the last five years up to 2023. While this ensures the inclusion of the most recent research, it also means that older, potentially relevant studies were not considered. Thirdly, we limited our search to PubMed as database. Although this database covers a wide range of relevant literature, there is a possibility that studies indexed in other databases were missed. Additionally, the inclusion/exclusion criteria, which we used to select studies, i.e. considering the type of article, type of reported outcome, type of reported healthcare setting, and type of reported instruments, might have excluded studies with relevant insights from the broader context of patient satisfaction assessment. Lastly, the entire process of search, data extraction, and synthesis was conducted by only one author (DR). This lack of validation by a second reviewer could have introduced biases or errors in the study selection and data extraction processes.

SECTION	ITEM	PRISMA-ScR CHECKLIST ITEM	REPORTED IN SECTION
Conclusions	21	Provide a general interpretation of the results with respect to the review questions and objectives, as well as potential implications and/or next steps.	Our review indicates that there is substantial research on patient satisfaction. We identified a significant number of instruments measuring patient satisfaction, yet there remains variability in their usage, reliability, and integration feasibility. Our objective was to identify a reliable and widely accepted instrument for measuring patient satisfaction that could be integrated into our comprehensive evaluation tool for digital health solutions following the results of the previous literature review and healthcare practitioner interviews. Among the instruments reviewed, the Patient Satisfaction Questionnaire – Short Form emerged as the most suitable due to its frequent use, demonstrated reliability, and compatibility with the dimensions identified in our previous review. Implications for future research include validating the combined instruments.
**FUNDING**			
Funding	22	Describe sources of funding for the included sources of evidence, as well as sources of funding for the scoping review. Describe the role of the funders of the scoping review.	As this research is part of a doctoral thesis at Witten/Herdecke University, it has not received any funding.
