# Supplementary material for: Service Quality Assessment of Digital Health Solutions in Outpatient Care: Qualitative Item Repository Development Study
Source: JMIR Form Res. 2025 Jul 24;9:e68276. doi: 10.2196/68276 (PMC12332462; doi:10.2196/68276)
Supplement: Multimedia Appendix 3 [file formative_v9i1e68276_app3.pdf]

Multimedia Appendix 3: COREQ Checklist – Healthcare Practitioner Interviews.

| DOMAIN | ITEM | GUIDE QUESTIONS / DESCRIPTIONS | REPORTED IN SECTION |
|---|---|---|---|
| **DOMAIN 1: RESEARCH TEAM AND REFLEXIVITY** | | | |
| ***Personal characteristics*** | | | |
| Interviewer/facilitator | 1 | Which author/s conducted the interview or focus group? | DR conducted the interviews. |
| Credentials | 2 | What were the researcher's credentials? (e.g., PhD, MD) | DR holds a Bachelor and a Master of Science in Biochemistry. |
| Occupation | 3 | What was their occupation at the time of the study? | DR is a Ph.D. candidate. |
| Gender | 4 | Was the researcher male or female? | DR is male. |
| Experience and training | 5 | What experience or training did the researcher have? | DR was experienced in conducting interviews based on his professional background in strategy consulting. |
| ***Relationship with participants*** | | | |
| Relationship established | 6 | Was a relationship established prior to study commencement? | There was no relationship established to the participants prior to the study. |
| Participant knowledge of the interviewer | 7 | What did the participants know about the researcher? (e.g., personal goals, reasons for doing the research) | In the beginning of the interview, participants were informed about the educational and professional background of the researcher, and the overall objectives and aims of the research and the PhD project. |
| Interviewer characteristics | 8 | What characteristics were reported about the interviewer/facilitator? (e.g., bias, assumptions, reasons, and interests in the research topic) | Participants were informed that the interest in the research topic was based on the researcher's professional background and the PhD project. |
| **DOMAIN 2: STUDY DESIGN** | | | |
| ***Theoretical framework*** | | | |
| Methodological orientation and theory | 9 | What methodological orientation was stated to underpin the study? (e.g., grounded theory, discourse analysis, ethnography, phenomenology, content analysis) | see *'Overview'* and *'HCP Interviews'* |
| ***Participant selection*** | | | |
| Sampling | 10 | How were participants selected? (e.g., purposive, convenience, consecutive, snowball) | see *'HCP Interviews'* |
| Method of approach | 11 | How were participants approached? (e.g., face-to-face, telephone, mail, email) | see *'HCP Interviews'* |
| Sample size | 12 | How many participants were in the study? | see *'HCP Interviews'* |
| Non-participation | 13 | How many people refused to participate or dropped out? Reasons? | We approached 111 healthcare practitioners, of which 83 did not respond to our interview enquiry, 12 declined to be interviewed due to a |

| DOMAIN | ITEM | GUIDE QUESTIONS / DESCRIPTIONS | REPORTED IN SECTION |
|---|---|---|---|
| | | | lack of time and high workload, 5 scheduled interviews were cancelled due to data saturation. |
| ***Setting*** | | | |
| Setting of data collection | 14 | Where was the data collected? (e.g., home, clinic, workplace) | see *'HCP Interviews'* |
| Presence of non-participants | 15 | Was anyone else present besides the participants and researchers? | see *'HCP Interviews'* |
| Description of sample | 16 | What are important characteristics of the sample? (e.g., demographic data, date) | see *'HCP Interviews'* |
| ***Data collection*** | | | |
| Interview guide | 17 | Were questions, prompts, guides provided by the authors? Was it pilot tested? | see *'HCP Interviews'*<br><br>We utilized a semi-structured interview guide for the interviews to allow for flexibility yet achieve standardization of the interview procedure. The interview guide was reviewed and approved by the ethics committee of the Universität Witten/Herdecke. |
| Repeat interviews | 18 | Were repeat interviews carried out? If yes, how many? | No repeat interviews were carried out. |
| Audio/visual recording | 19 | Did the research use audio or visual recording to collect the data? | see *'HCP Interviews'* |
| Field notes | 20 | Were field notes made during and/or after the interview or focus group? | No field notes were made during the interviews. However, a short summary of the interview was written after transcription. |
| Duration | 21 | What was the duration of the interviews or focus group? | see *'HCP Interviews'* |
| Data saturation | 22 | Was data saturation discussed? | see *'HCP Interviews'*<br><br>We planned to interview between 10 and 15 healthcare practitioners based on data saturation. This was achieved, i.e., no new content on top of the derived themes based on the literature research emerged by the time we interviewed 11 healthcare practitioners. |
| Transcripts returned | 23 | Were transcripts returned to participants for comment and/or correction? | We did not return transcripts to participants. |
| **DOMAIN 3: ANALYSIS AND FINDINGS** | | | |
| ***Data analysis*** | | | |
| Number of data coders | 24 | How many data coders coded the data? | DR coded the data. |

| DOMAIN | ITEM | GUIDE QUESTIONS / DESCRIPTIONS | REPORTED IN SECTION |
|---|---|---|---|
| Description of the coding tree | 25 | Did authors provide a description of the coding tree? | see *'HCP Interviews'* |
| Derivation of themes | 26 | Were themes identified in advance or derived from the data? | see *'HCP Interviews'* |
| Software | 27 | What software, if applicable, was used to manage the data? | see *'HCP Interviews'* |
| Participant checking | 28 | Did participants provide feedback on the findings? | We did not ask for feedback on the findings from participants. |
| ***Reporting*** | | | |
| Quotations presented | 29 | Were participant quotations presented to illustrate the themes/findings? Was each quotation identified? (e.g., participant number) | We do not present participant quotes. |
| Data and findings consistent | 30 | Was there consistency between the data presented and the findings? | Data presented and findings are consistent. |
| Clarity of major themes | 31 | Were major themes clearly presented in the findings? | see *'HCP Interviews'*<br><br>We do not discriminate between major or minor themes. |
| Clarity of minor themes | 32 | Is there a description of diverse cases or discussion of minor themes? | see *'HCP Interviews'* |
