# Supplementary material for: Service Quality Assessment of Digital Health Solutions in Outpatient Care: Qualitative Item Repository Development Study
Source: JMIR Form Res. 2025 Jul 24;9:e68276. doi: 10.2196/68276 (PMC12332462; doi:10.2196/68276)
Supplement: Multimedia Appendix 4 [file formative_v9i1e68276_app4.pdf]

Multimedia Appendix 4: Translated Semi-structure Interview Guide.

Introduction____

_

Introduction of the interviewer

Introduction of the overarching research topic

Introduction of the interview topic and format

Introduction of relevant definitions (i.e., digital health solution, healthcare service quality)

Part 1: Experience with digital health solutions______________________________________

- Could you tell me a little about yourself?
  (E.g., gender, age, profession, professional experience, employment status)
- Could you tell me a little about the practice you are working in?
  (E.g., therapeutic area, location, type of practice, type of patients, size and composition of team)
- Do you have any experience with digital health solutions (e.g. video consultations, electronic patient records, appointment booking platforms, digital anamnese)?
  - If yes, which digital health solutions have you implemented in your practice and how often do you use them?
  - If yes, how involved were you in the selection or implementation process of the digital health solution?

Part 2: Healthcare service quality dimensions in outpatient care_______________________

- How would you rate the healthcare service quality of your practice on a scale from 1 (low quality) to 5 (high quality)? Which criteria did you consider to come to this conclusion?
- What other dimensions do you consider relevant in relation to the healthcare service quality of general and specialist practices?

- *(If digital health solutions are used):* In your experience, which mentioned dimensions of healthcare service quality are affected by digital health solutions?
- *(If involved in selection or implementation process):* Which dimensions have played a role in the selection and/or implementation of digital health solutions?
- On a scale from 1 (not at all relevant) to 10 (very relevant), how would you rate the relevance of the following dimensions for the healthcare service quality of general and specialist practices?
    a. Empathy
    b. Tangibles
    c. Safety
    d. Efficiency
    e. Improvement of care services
    f. Accessibility
    g. Information

    (Comment: A short definition of each dimensions was provided)
- For which of these dimension did you observe or do you expect effects on healthcare service quality in general and sepcialist practices?
- Are there any other dimensions with regard to healthcare service quality we did not discuss yet?

Closing

Conclusion

Open questions and feedback

Thank you for participation and goodbye
