# Supplementary material for: Service Quality Assessment of Digital Health Solutions in Outpatient Care: Qualitative Item Repository Development Study
Source: JMIR Form Res. 2025 Jul 24;9:e68276. doi: 10.2196/68276 (PMC12332462; doi:10.2196/68276)
Supplement: Multimedia Appendix 5 [file formative_v9i1e68276_app5.pdf]

Table S4: Development of the DigiHEALTHQUAL Questionnaire; Original dimensions and items from HEALTHQUAL, OPEQ, and PSQ-18 followed by required adaptations and de-duplication for the construction of DigiHEALTHQUAL.[a]

**Original instruments**				**Consolidation & refinement**		**Derived instrument**		
**Instrument**	**Dimension**	**Item ID**	**Item**	**Type of change**	**Comment**	**Adjusted item**	**Consolidated dimension**	**Comment**
HEALTHQUAL	Efficiency	H_EF1	Attitudes about not using unnecessary medication	Keep		The practice team strives to avoid unnecessary medications.	Efficiency	
HEALTHQUAL	Efficiency	H_EF2	Degree of efforts for providing appropiate treatment methods	Keep		The practice team strives to find the most appropriate treatment method for their patients.	Efficiency	
HEALTHQUAL	Efficiency	H_EF3	Reasonable medical expenses	Out	Adjustment to account for major differences in national healthcare systems	N/A	N/A	
HEALTHQUAL	Efficiency	H_EF4	Appropriateness of cost for the medical services provided	Out	Adjustment to account for major differences in national healthcare systems	N/A	N/A	
HEALTHQUAL	Efficiency	H_EF5	Degree of convenience for treatment procedures	Keep		The practice team makes the treatment procedures comfortable for the patients.	Efficiency	
HEALTHQUAL	Efficiency	H_EF6	Degree of efforts for reducing unnecessary procedures	Keep		The practice team strives to avoid unnecessary treatments and tests.	Efficiency	
HEALTHQUAL	Empathy	H_EM1	Polite attitude of employees	Keep		The practice team shows a polite attitude towards the patients.	Empathy	
HEALTHQUAL	Empathy	H_EM2	Explaining the details	Keep		The practice team takes the time to explain the details to the patients.	Empathy	
HEALTHQUAL	Empathy	H_EM3	Listen to the patient	Keep		The practice team takes the time to listen to the patients.	Empathy	
HEALTHQUAL	Empathy	H_EM4	Understand and consider the patient's situation	Keep		The practice team understands and considers the patient's situation.	Empathy	
HEALTHQUAL	Empathy	H_EM5	A sense of closeness and friendliness	Keep		The interactions of the practice team with the patients are characterized by closeness and friendliness.	Empathy	
HEALTHQUAL	Empathy	H_EM6	Hospital knows what the patient wants	Adjusted	Adjusted to broader outpatient setting	The practice team understands what the patients want.	Empathy	
HEALTHQUAL	Empathy	H_EM7	Hospital understands the patient's problems as empathy	Adjusted	Adjusted to broader outpatient setting	The practice team empathizes with the patients' problems.	Empathy	
HEALTHQUAL	Improvements of care service	H_IM1	Appropriateness of care service provided	Keep		The medical treatment is perceived as appropriate.	Improvements of care service	
HEALTHQUAL	Improvements of care service	H_IM2	Recognition and efforts for the best treatment by the medical staff	Keep		The practice team makes great efforts for the best possible care of the patients.	Improvements of care service	
HEALTHQUAL	Improvements of care service	H_IM3	Improvement in medical condition as a result of efforts and treatment	Keep		The patients' medical conditions improve through the treatment in the practice.	Improvements of care service	
HEALTHQUAL	Improvements of care service	H_IM4	Degree of improved patient condition after using this hospital care	Adjusted	Adjusted to broader outpatient setting	The overall condition of the patients improves through the visit to the practice.	Improvements of care service	
HEALTHQUAL	Improvements of care service	H_IM5	Degree of explanation to the patient to prevent related diseases	Keep		The practice team strives to prevent related diseases through patient education.	Improvements of care service	
HEALTHQUAL	Improvements of care service	H_IM6	Degree of efforts and willingness to prevent disease	Keep		The practice team is committed to supporting the patients in preventing a recurrence of illness.	Improvements of care service	
HEALTHQUAL	Improvements of care service	H_IM7	Improvement of disease through this hospital's treatment	Adjusted	Adjusted to broader outpatient setting	The patients' conditions improve through the treatment in this practice.	Improvements of care service	
HEALTHQUAL	Improvements of care service	H_IM8	Degrees of disease prevention to communities	Out	Adjustment to account for major differences in national healthcare systems	N/A	N/A	
HEALTHQUAL	Safety	H_SA1	Degree of a comfortable and safe environment for receiving treatment	Keep		The practice team creates a comfortable and safe environment for the treatment of the patients.	Safety	
HEALTHQUAL	Safety	H_SA2	Degree of the feeling that doctors would not make misdiagnoses	Keep		The patients trust the doctors not to make misdiagnoses.	Safety	

HEALTHQUAL	Safety	H_SA3	Degree of feeling that nurses would not make mistakes	Keep		The patients trust the medical assistants not to make mistakes.	Safety	
HEALTHQUAL	Safety	H_SA4	Degree of confidence about medical proficiency of this hospital	Adjusted	Adjusted to broader outpatient setting	The patients trust the medical proficiency of the practice team.	Safety	
HEALTHQUAL	Safety	H_SA5	Degree of a hospital environment that is safe from infection	Adjusted	Adjusted to broader outpatient setting	The patients feel safe from infections in the practice.	Safety	
HEALTHQUAL	Safety	H_SA6	Degree of a comfortable and safe environment for patients	Keep		The practice team creates a pleasant and safe atmosphere in all interactions with the patients.	Safety	
HEALTHQUAL	Tangible	H_TA1	Degree of securing advanced medical equipment	Adjusted	Adjustment to include DHS	The practice uses modern medical devices and digital health solutions.	Tangible	
HEALTHQUAL	Tangible	H_TA2	Degree of securing medical staff with advanced skills and knowledge	Keep		The members of the practice team possess advanced skills and knowledge.	Tangible	
HEALTHQUAL	Tangible	H_TA3	Degree of convenient facilities	Adjusted	Adjustment to include DHS	The patient experience is comfortable both in the practice and online.	Tangible	
HEALTHQUAL	Tangible	H_TA4	Degree of cleanliness of employee uniforms	Adjusted	Adjustment to include DHS	The practice team always makes a clean and tidy impression in interactions with the patients both in the practice and online.	Tangible	
HEALTHQUAL	Tangible	H_TA5	Overall cleanliness of the hospital	Adjusted	Adjustment to include DHS	The overall impression that patients get of the practice, both in the practice rooms and online, is clean and tidy.	Tangible	
OPEQ	Clinic access	O_CA1	Ease of finding clinic/ward	Adjusted	Adjustment to include DHS	The practice and/or its online information is easy for patients to find.	Accessibility	
OPEQ	Clinic access	O_CA2	Ease of finding way within clinic/ward	Adjusted	Adjustment to include DHS	It is easy for patients to navigate the online offerings and/or within the practice.	Accessibility	
OPEQ	Communication	O_CO1	Enough time for dialogue	Keep		The practice team takes enough time for the conversation with the patients.	Empathy	Mapped to "Empathy"
OPEQ	Communication	O_CO2	Person understandable	Keep		The members of the practice team are perceived as understandable.	Empathy	Mapped to "Empathy"
OPEQ	Communication	O_CO3	Person competent	Out	Redundant with H_TA2	N/A	N/A	
OPEQ	Communication	O_CO4	Person caring	Out	Redundant with H_EM5	N/A	N/A	
OPEQ	Communication	O_CO5	Opportunity to give sufficient information	Out	Redundant with H_EM2	N/A	N/A	
OPEQ	Communication	O_CO6	(Left with) Unanswered questions	Out	Redundant with H_EM2	N/A	N/A	
OPEQ	Hospital standards	O_HS1	Waiting room	Out	Redundant with H_TA5	N/A	N/A	
OPEQ	Hospital standards	O_HS2	Toilet	Out	Redundant with H_TA5	N/A	N/A	
OPEQ	Hospital standards	O_HS3	Cleanliness	Out	Redundant with H_TA5	N/A	N/A	
OPEQ	Organization	O_OR1	Background information available	Adjusted	Adjustment to include DHS	The practice team provides sufficient background information to the patients both in the practice and online.	Efficiency	Mapped to "Efficiency"
OPEQ	Organization	O_OR2	Staff collaboration (good)	Keep		The practice team works well together.	Efficiency	Mapped to "Efficiency"
OPEQ	Organization	O_OR3	Organization of work	Keep		The work in the practice is well organized.	Efficiency	Mapped to "Efficiency"
OPEQ	Organization	O_OR4	Person well prepared	Keep		The practice team enters the interaction with the patients well prepared.	Efficiency	Mapped to "Efficiency"
OPEQ	Pre-visit communication	O_PC1	Acceptability of appointment waiting time	Keep		The waiting time for appointments is acceptable for the patients.	Accessibility	Merged with "Accessibility"
OPEQ	Pre-visit communication	O_PC2	Information from clinic received in advance	Keep		The patients receive all necessary information in advance.	Accessibility	Merged with "Accessibility"
OPEQ	Pre-visit communication	O_PC3	Ease of accessing clinic staff	Keep		The practice team is easily accessible to the patients.	Accessibility	Merged with "Accessibility"
OPEQ	(Quality of) Information	O_QI1	Information Self-care	Keep		The patients receive information on self-care from the practice team.	Information	
OPEQ	(Quality of) Information	O_QI2	Information Medication/Side-effects	Keep		The patients receive information on medication and their side effects from the practice team.	Information	
OPEQ	(Quality of) Information	O_QI3	Information Examinations	Keep		The patients receive information on planned examinations from the practice team.	Information	
OPEQ	(Quality of) Information	O_QI4	Information Examination/Test results	Keep		The patients receive information on the results of examinations and tests from the practice team.	Information	
OPEQ	(Quality of) Information	O_QI5	Information Condition/Prognosis	Keep		The patients receive information on their condition and prognosis from the practice team.	Information	

OPEQ	(Quality of) Information	O_QI6	Consulted about examination/treatment	Keep		The patients are consulted about planned examinations and treatments by the practice team.	Information	
PSQ-18	Accessibility & Convenience	P_AC1	I have easy access to the medical specialists I need	Out	Redundant with O_PC3	N/A	N/A	
PSQ-18	Accessibility & Convenience	P_AC2	Where I get medical care, people have to wait too long for emergency treatment	Keep		Patients with urgent concerns have to wait too long for their treatment.	Accessibility	Merged with "Accessibility"
PSQ-18	Accessibility & Convenience	P_AC3	I find it hard to get an appointment for medical care right away	Out	Redundant with O_PC1	N/A	N/A	
PSQ-18	Accessibility & Convenience	P_AC4	I am able to get medical care whenever I need it	Out	Redundant with O_PC1	N/A	N/A	
PSQ-18	Communication	P_CO1	Doctors are good about explaining the reason for medical tests	Out	Redundant with H_EM2 and O_QI3	N/A	N/A	
PSQ-18	Communication	P_CO2	Doctors sometimes ignore what I tell them	Out	Redundant with H_EM6	N/A	N/A	
PSQ-18	Financial aspects	P_FA1	I feel confident that I can get the medical care I need without being set back financially	Out	Adjustment to account for major differences in national healthcare systems	N/A	N/A	
PSQ-18	Financial aspects	P_FA2	I have to pay for more of my medical care than I can afford	Out	Adjustment to account for major differences in national healthcare systems	N/A	N/A	
PSQ-18	General satisfaction	P_GS1	The medical care I have been receiving is just about perfect	Keep		The patients perceive the treatment overall as ideal.	General satisfaction	
PSQ-18	General satisfaction	P_GS2	I am dissatisfied with some things about the medical care I receive	Keep		The patients are dissatisfied with some aspects of their treatment.	General satisfaction	
PSQ-18	Interpersonal Manner	P_IM1	Doctors act too businesslike and impersonal towards me	Out	Redundant with H_EM5	N/A	N/A	
PSQ-18	Interpersonal Manner	P_IM2	My doctors treat me in a very friendly and courteos manner	Out	Redundant with H_EM5	N/A	N/A	
PSQ-18	Technical quality	P_TQ1	I think my doctor's office has everything needed to provide complete medical care	Out	Redundant with H_TA1	N/A	N/A	
PSQ-18	Technical quality	P_TQ2	Sometimes doctors make me wonder their diagnosis is correct	Out	Redundant with H_SA2	N/A	N/A	
PSQ-18	Technical quality	P_TQ3	I have some doubts about the ability of the doctors who treat me	Out	Redundant with H_TA2	N/A	N/A	
PSQ-18	Technical quality	P_TQ3	When I go for medical care, they are careful to check everything when treating and examining me	Keep		The practice team takes enough time to check everything when examining and treating the patients.	Safety	Mapped to "Safety"
PSQ-18	Time spent with doctor	P_TS1	Those who provide my medical care sometime hurry too much when they treat me	Out	Redundant with O_CO1	N/A	N/A	
PSQ-18	Time spent with doctor	P_TS2	Doctors usually spend plenty of time with me	Out	Redundant with O_CO1	N/A	N/A	
N/A	N/A	N/A	N/A	Added	Created question similar to H_SA5 to cover new aspects of DHS	The patients feel that their personal data is stored and processed safely in the practice.	Safety	

[a]74 items were derived from three instruments (HEALTHQUAL, OPEQ, PSQ-18); 24 items were removed (duplicates, adjustment to healthcare system compatibility), 13 items were adjusted to the focus of this study (outpatient setting, effect of DHS), one item was added; the resulting DigiHEALTHQUAL instrument contains 51 items across eight dimensions; DHS = Digital health solution, OPEQ = outpatient experience questionnaire, PSQ-18 = Patient satisfaction questionnaire-Short form.
